# The Effect of a Diet Moderately High in Protein and Fiber on Insulin Sensitivity Measured Using the Dynamic Insulin Sensitivity and Secretion Test (DISST)

**DOI:** 10.3390/nu9121291

**Published:** 2017-11-27

**Authors:** Lisa Te Morenga, Paul Docherty, Sheila Williams, Jim Mann

**Affiliations:** 1 Department of Human Nutrition, University of Otago, PO Box 56, Dunedin 9054, New Zealand; jim.mann@otago.ac.nz; 2 Edgar Diabetes and Obesity Research Centre, University of Otago, Dunedin 9054, New Zealand; 3 Riddet Institute, University of Otago, PO Box 56, Dunedin 9054, New Zealand; 4 Department of Mechanical Engineering, University of Canterbury, Christchurch 8140, New Zealand; paul.docherty@canterbury.ac.nz; 5 Department of Preventive and Social Medicine, University of Otago, Dunedin 9054, New Zealand; sheila.williams@otago.ac.nz

**Keywords:** diet, dietary protein, dietary fibre, insulin sensitivity assessment, insulin sensitivity, insulin resistance, metabolic syndrome

## Abstract

Evidence shows that weight loss improves insulin sensitivity but few studies have examined the effect of macronutrient composition independently of weight loss on direct measures of insulin sensitivity. We randomised 89 overweight or obese women to either a standard diet (StdD), that was intended to be low in fat and relatively high in carbohydrate (*n* = 42) or to a relatively high protein (up to 30% of energy), relatively high fibre (>30 g/day) diet (HPHFib) (*n* = 47) for 10 weeks. Advice regarding strict adherence to energy intake goals was not given. Insulin sensitivity and secretion was assessed by a novel method—the Dynamic Insulin Sensitivity and Secretion Test (DISST). Although there were significant improvements in body composition and most cardiometabolic risk factors on HPHFib, insulin sensitivity was reduced by 19.3% (95% CI: 31.8%, 4.5%; *p* = 0.013) in comparison with StdD. We conclude that the reduction in insulin sensitivity after a diet relatively high in both protein and fibre, despite cardiometabolic improvements, suggests insulin sensitivity may reflect metabolic adaptations to dietary composition for maintenance of glucose homeostasis, rather than impaired metabolism.

## 1. Introduction

There is considerable evidence to show that weight loss can improve insulin sensitivity and reduce the risk of diabetes and cardiovascular disease (CVD) [1,2]. Few studies, however, have examined the effect of diet intervention on direct measures of insulin sensitivity (IS), particularly in the context of weight maintenance. However, there is a substantial body of literature to show that variations in the macronutrient composition of diets can modify the cardiometabolic abnormalities associated with insulin resistance (IR) and therefore reduce the risk of diabetes. Low fat, high carbohydrate diets have been shown to raise triglyceride (TG) levels and to reduce high density lipoprotein (HDL) concentrations [3] whereas carbohydrate restriction may have the opposite effect, while also having a positive influence of promoting low density lipoprotein (LDL) particle size [4]. High protein diets have become a popular approach to weight loss and improving body composition, with increasing evidence showing modest benefits over standard protein, relatively high carbohydrate diets, at least in the relatively short term (one to two years) [5]. An alternative approach to reducing the metabolic abnormalities associated with low fat, high carbohydrate diets is to modify the quality of the carbohydrate consumed. There is some experimental and much epidemiological evidence to suggest that high fibre (particularly soluble fibre), low glycemic index (GI) carbohydrates, derived from minimally processed wholegrain cereals, fruit, vegetables and legumes may improve insulin sensitivity, maintain glucose homeostasis, reduce postprandial insulin concentrations, and reduce blood pressure in comparison with refined and high GI carbohydrates [6].

The gold-standard method for assessing insulin sensitivity is the hyperinsulinemic euglycaemic clamp [7]. However the clamp is rarely used, as it is technically difficult and time-consuming, and because highly trained clinicians are required to perform the test, it is expensive to conduct [8]. Numerous techniques have been developed to simplify the measurement of IS but this has been at the expense of reliability and reproducibility [7]. Therefore, researchers have more frequently used surrogate indices of IS, based on fasting blood parameters, such as the homeostatic model of assessment (HOMA) [9] and the McAuley [10] methods. Such methods have been developed to describe the relationship of IS to disease progression in longitudinal population studies, but relatively poor reproducibility and reliability limits their usefulness for detecting dietary-induced changes in IS, in clinical studies [8]. The Dynamic Insulin Sensitivity and Secretion Test (DISST) was developed to address these issues [11]. The DISST is a variation of the infrequently sampled, low dose insulin-modified intravenous glucose tolerance test (IM-IVGTT) that can simultaneously provide highly reproducible estimates of IS and beta cell function [11]. 

The aim of this study was to examine the effect of a reduced carbohydrate diet that was moderately high in both protein and dietary fiber (HPHFib diet) on IS and insulin secretion, using the DISST method, in overweight and obese women at risk of diabetes, independently of weight loss.

## 2. Materials and Methods 

### 2.1. Subjects and Experimental Protocol

The subjects, study design and diets have been previously described [12]. In brief, 89 women at risk of diabetes, who met screening criteria, were randomly assigned to either a standard diet (StdD) or a relatively high protein and high fibre diet (HPHFib) for 10 weeks. Women on medications to treat metabolic syndrome risk factors (raised blood pressure, raised lipids or abnormal glucose control) were not excluded as the aim was to recruit a cohort who were likely to be insulin resistant and at high risk of diabetes. Advice regarding strict adherence to energy intake goals was not given (i.e., total energy intakes were ad libitum). Eighty-nine women were randomly assigned to treatment, 6 withdrew before receiving the allocated treatment, 8 withdrew during the treatment period and 75 women completed the entire study.

At baseline, week 4 and week 10, measurements of height, weight, waist circumference and seated blood pressure were taken and fasting blood samples were collected for the measurement of serum lipids, and plasma glucose and insulin (in vacutainers containing antiglycolytic and/or EDTA anticoagulants). A DISST test was then carried out. Body composition was measured by dual x-ray absorptiometry (DXA) at baseline and week 10 only. 

Each subject gave informed, written consent and all experimental procedures were approved by the University of Otago Human Ethics Committee (reference 06/182). The trial was registered with the Australian New Zealand Clinical Trials Registry (ACTRN12607000154404).

### 2.2. Diets

The standard diet (StdD) group received dietary advice based on the New Zealand Food and Nutrition Guidelines for Healthy Adults [13]. Dietary goals were for approximately 20% of total energy (TE) to be derived from protein, 50% from carbohydrate and 30% from total fat with <10% from saturated fat. Dietary fibre intake was to be ≥25 g/day. The StdD group were given a resource available from the Ministry of Health in New Zealand, designed to facilitate adherence to these guidelines, and a food group checklist which provided daily serving targets for major food groups. Tailored dietary advice based on these recommendations was delivered by the researcher (LTM) during individual 45–60 min counselling sessions at week 0 and week 5. Participants were encouraged to make fortnightly appointments to monitor progress and discuss strategies for maintaining adherence to the diet, but this was not compulsory.

The HPHFib diet was designed to achieve 30% TE from protein, 50% from carbohydrate, 20% from fat, and a dietary fibre intake of ≥35 g/day. Individuals were asked to increase their usual protein intake with lean meats, fish or low-fat dairy foods and to choose carbohydrates that were particularly high in soluble fibre, such as oats, certain legumes, nuts, dried fruit and stone fruits as well as wholegrain breads and cereals. Restriction of fat intake was necessary, in order to achieve the dietary fibre and protein goals, without increasing energy intakes or requiring a dietary fibre supplement. Consumption of refined carbohydrates including white bread, white rice, pasta, cakes, biscuits and scones was discouraged. The HPHFib group was given material especially prepared for this study, including recipes and sample diet plans, since relevant material was not readily available. Because the HPHFib diet would not have been familiar to the typical New Zealander, participants were optionally provided with a variety of pre-prepared frozen main course meals, especially formulated to be high in protein and fibre as well as 30 g/day high protein whey concentrate powder (NZMP™ Whey Protein Concentrate 392, Fonterra Co-operative Group Limited, New Zealand), wholegrain breakfast cereal and bread, canned beans and canned fish. The high protein whey powder was supplied to enable participants to maintain a relatively conventional eating plan, based around a cereal-based breakfast, while also increasing protein intake. Tailored dietary advice based on these recommendations was delivered by the researcher (LTM) during individual 45–60 min counselling sessions at week 0 and week 5. Participants met with the researcher on a weekly basis in subsequent weeks to monitor progress and discuss strategies for maintaining adherence to the diet. Thus, the HPHFib group was provided with a more intensive treatment than the StdD group. 

For both groups, during the initial 4-week study phase, advice was given regarding food choices necessary to achieve the required macronutrient composition while maintaining usual weight. This was to allow us to examine whether dietary composition influences insulin sensitivity independently of weight loss. During the following 6 weeks, they were encouraged to continue the recommended dietary pattern, but were not required to maintain their baseline weight; that is, they could reduce their food intake if they had a reduced appetite as a result of their dietary changes. This was a pragmatic decision in recognition that overweight women volunteering for dietary intervention studies will be motivated to reduce their weight and that it could be challenging to maintain energy intake on the HPHFib diet because of the increased volume of food.

Participants completed a weighed 3-day diet record, including 2 non-consecutive weekdays and one weekend day, prior to commencing the intervention and at week 8. Dietary intakes of various nutrients were calculated using the Diet Cruncher for Macintosh V1.2.0 program (Waydown South Software, Dunedin, New Zealand) using FOODFiles, the New Zealand food composition database [14]. Missing food items were obtained from manufacturers’ information or other published references.

### 2.3. Insulin Sensitivity Measurements

Insulin sensitivity and other risk factors associated with the metabolic syndrome were assessed at baseline, week 4 and week 10. Insulin sensitivity was assessed with the DISST method, conducted by a research nurse, under medical supervision. After a 10–12 h fast, participants had a cannula inserted into the antecubital fossa. Blood samples were drawn at *t* = 0, 10, 15, 25, and 35 min, for measurement of plasma glucose, and serum insulin and C-peptide. A 10 g bolus of intravenous glucose was given at *t* = 5 min, and 1 U of Actrapid insulin was given immediately after the *t* = 15-min sample.

The DISST model estimates insulin sensitivity (SI), glucose distribution volume (Vg), and first-pass (xL) and subsequent hepatic insulin clearance (nL) and three metrics of β-cell function, derived from insulin production profiles and C-peptide data [11], following the methods of Van Cauter et al [15,16]. The basal rate (Ub) indicates the rate of insulin production that an individual requires to maintain blood glucose at fasting levels. The area under the curve (AUC10) measures the first-phase insulin production, produced immediately after the glucose bolus. AUC2nd measures the individual’s second phase of insulin production during the 20 min after the period measured by AUC10 [11].

### 2.4. Laboratory Analyses

Whole blood samples were centrifuged at 1650 g for 15 min; then, samples were pipetted into polyethylene cryovials and stored at −80 °C. Laboratory results, at all time-points, for all subjects, were performed in batch, within the same assay. Serum insulin and C-peptide were measured using a specific insulin electrochemiluminescence immunoassay (ECLIA) (Roche, Cat. No. 12017547) for the Elecsys® analyzer (Roche Diagnostics, Mannheim, Germany), with a coefficient of variation of 1.5%. Serum total cholesterol (total-cholesterol and triglycerides (TG) concentrations were measured enzymatically with Roche kits and calibrators on a Cobas Mira analyzer, as was plasma glucose (Roche Hexokinase Cat. No. 11447513216). Coefficients of variation were 2.8% for total-cholesterol, 4.4% for TG and 0.5% for plasma glucose. HDL-cholesterol was measured in the supernatant after precipitation of apolipoprotein B containing lipoproteins with phosphotungstate/magnesium chloride solution [17] with a coefficient of variation of 3.6%. LDL-cholesterol was calculated using the Friedewald equation: (total – cholesterol − HDL-cholesterol − (TG/2.18)) [18].

### 2.5. Analysis and Statistics

Insulin resistance was estimated by HOMA-IR index, using the HOMA-IR2 calculator [9] and by the McAuley index, using fasting insulin and TG, where predicted insulin sensitivity is expressed as an exponent (2.63 − 0.28 ln(fasting insulin) − 0.31 ln(fasting TG)) [10]. A McAuley index value ≤ 6.3 indicated insulin resistance.

The number of participants required to detect a 30% difference in insulin resistance, as assessed by the HOMA-IR index, with 80% power, at a level of significance of 0.05, was 72. Statistical analyses were performed using the STATA statistical software package 9.0 (Stata, College Station, TX, USA). Baseline data are presented as mean (SD). Elsewhere, data are presented as mean (SE) or as geometric means (min, max) for logarithmically transformed values. Data were analysed on a modified intention-to-treat basis (i.e., without imputation of missing values), using a mixed model for repeated measures, with “participant” as a random effect of the treatment over the two intervention phases (weeks 0–4 and weeks 5–10) [19] and baseline values as a covariate [20] (overall model). Interactions between time and diet were tested by including a term for “time × diet” in the model. Since there were significant interactions between time and diet for some biochemical variables, the effects of treatment at week 4 and week 10 were estimated separately by analysis of covariance, using baseline values as a covariate. The overall model is also presented for variables where there was no significant “time × diet” interaction. For dietary variables, only the overall model is shown. This does not change the interpretation of the results, but has a conservative effect on statistical power since the number of observations is decreased and the SE is increased with the separate analyses.

The estimates for all variables, except for those relating to body composition, have been further adjusted for baseline weight and change in weight during the intervention period. This is because the intention of the study was to assess the effect of macronutrient composition on insulin sensitivity, independently of weight loss. Both unadjusted and adjusted models are presented.

A post-hoc analysis was conducted to examine the DISST insulin sensitivity and insulin secretion models for interactions between insulin sensitivity status and diet group, by including an interaction term for group × insulin resistance status. ISI_clamp equivalent_ < 1.0 e^−2^ mg/kg/(pmol/L)/min was used to define insulin resistance.

## 3. Results

Table 1 presents the baseline characteristics of the participants who started the dietary intervention. More participants were initially randomised to the HPHFib group (44 vs. 39), they were slightly older and had a higher estimated prevalence of insulin resistance. There were 39 women in the HPHFib group and 37 women in the standard diet group, for whom clinical and anthropometric data are reported. 

Baseline dietary macronutrient intakes were well matched (Table 2). Participants on the HPHFib diet consumed significantly more protein and dietary fibre and less total fat and saturated fat than the StdD group during the study, but there was no difference in total carbohydrate intake. Of the increase in total dietary fibre, 38% was soluble fibre. On average, HPHFib participants consumed 10.1 (SE 13.5) g/day of whey protein powder providing 7.6 g/day protein. Legumes, lean mean and chicken and fish provided the additional protein consumed by the HPHFib group. Reported energy intakes declined over the 10 weeks in both the control and HPHFib groups but there was no evidence of a difference between the two groups. 

Although the first 4 weeks of the study were intended to achieve weight maintenance, at week 4, the HPHFib participants had lost a small amount of weight, whereas the StdD participants had not changed. The difference between the groups was statistically significant. There was no further weight loss in the second phase of the study. Body composition was only measured at baseline and week 10. Total fat mass and truncal fat mass were lower in HPHFib than in StdD. Lean mass did not change in either group and there was no difference in waist circumference (Table 3). 

In the unadjusted models, DISST insulin sensitivity was reduced in the HPHFib group, relative to the StdD group, at both week 4 and week 10, but the difference between diets did not reach statistical significance (i.e., *p* < 0.05) (Table 4). When DISST insulin sensitivity was adjusted for weight loss, there was trend towards decreased insulin sensitivity at week 4 in the HPHFib group and a statistically significant decrease in insulin sensitivity at week 10. There was a statistically significant reduction in basal insulin secretion (U_b_) in the HPHFib group compared with the StdD group at week 10, although this was attenuated after adjustment for weight loss. In contrast, fasting glucose was significantly lower at week 10 on the HPHFib diet in the unadjusted model but this effect was attenuated in the model adjusted for weight loss. There was no evidence of differences in insulin concentrations, HOMA-IR2, the McAuley insulin sensitivity index and SBP between treatments (Table 5).

Post-hoc analyses testing for interaction effects between diet group, insulin sensitivity status and DISST metrics showed no significant differences between insulin sensitive and insulin resistant individuals (range of *p*-values: 0.084 to 0.723). This indicates that insulin sensitivity decreased in both insulin sensitive and insulin resistant individuals in the HPHFib group compared with those in the StdD group. 

There was also generally an improvement in lipid concentrations in the HPHFib group, whereas there was no change in the control group. Total cholesterol was lower at both week 4 and week 10, even after adjustment for weight loss and this was significantly different to the StdD group in whom total-cholesterol increased. LDL cholesterol was also reduced in the HPHFib group and the difference was significant at week 10 in the unadjusted model, although the effect was attenuated in the adjusted model. On the other hand, there was a small reduction in HDL cholesterol at week 4 in the HPHFib group but this had improved by week 10. TG was reduced in the HPHFib group and unchanged in the StdD group, but the difference was not significant (Table 6).

## 4. Discussion

This study showed that a modest increase in consumption of both dietary protein and fibre, without emphasis on energy reduction, improved several cardiometabolic risk factors in overweight women. There were modest reductions in body mass (1.2 kg), total body fat (1.0 kg) and central body fat (0.7 kg), with no loss of lean mass and improvements in total serum and LDL cholesterol concentrations. However, insulin sensitivity, as measured by DISST, declined throughout the 10-week study, even during the first 4 weeks, when moderate weight loss was achieved. 

Prospective analyses and lifestyle intervention studies indicate, with remarkable consistency, that high fibre diets are associated with a reduced risk of diabetes and CVD. Cross-sectional analyses of epidemiological studies, using static measures of insulin sensitivity, based on fasting concentrations of glucose and insulin, such as the HOMA-IR index, show a positive association between dietary fibre [21,22,23] or wholegrains [24,25] and insulin sensitivity. These findings are confirmed by cross sectional analyses, using dynamic insulin and glucose stimulated models of insulin sensitivity, including the frequently sampled intravenous glucose tolerance test [24] and the clamp [25]. Lifestyle intervention studies also provide evidence for a beneficial effect of dietary fibre from wholegrains, fruit and vegetables on insulin sensitivity [26,27] and reducing the risk of progression from impaired glucose tolerance (IGT) to diabetes [28,29,30]. However, the effect of dietary fibre cannot be disentangled from the effect of other changes made in conjunction with lifestyle improvement. 

Various mechanisms have been proposed as to how dietary fibre might influence insulin sensitivity, but there is as yet no definitive explanation. Refined carbohydrate foods are quickly absorbed and have hyperinsulinaemic and hyperglycaemic effects—both of which can lead to the down-regulation of GLUT 4 glucose transporters in peripheral tissues and a reduction in insulin sensitivity [31,32]. In contrast, intact or minimally processed high fibre carbohydrate foods and those with a low glycaemic index (GI) are typically more slowly digested and absorbed, resulting in reduced glycaemic and insulinaemic responses [33]. In addition, the fermentation of indigestible dietary fibre in the colon produces short-chain fatty acids, which may also regulate glucose homeostasis by inhibiting hepatic glucose production, stimulating hepatic glucose storage through glycogen synthesis and improving peripheral insulin sensitivity [34]. 

A relatively small number of clinical trials, involving healthy, overweight, insulin resistant and diabetic patients, have shown that wholegrain-rich, high fibre diets can improve insulin sensitivity and glucose metabolism using direct methods of assessing insulin sensitivity relative to diets with low-fibre diets or those based on refined grains [35,36,37,38], but the evidence is inconsistent [39]. Acute studies, comparing the effect of high fibre breads with lower fibre breads, are equally conflicting [40,41]. However, there is no evidence to suggest that high fibre diets impair insulin sensitivity. Studies examining the effect of GI on glucose homeostasis and direct measurements of insulin sensitivity have also not shown conclusive benefits of low GI diets compared with high GI diets. A carefully designed crossover study by Järvi and colleagues compared two identical diets differing only in GI in subjects with type 2 diabetes mellitus (T2DM) [42]. Insulin sensitivity, measured by the clamp, increased during both phases, but there was no effect of GI. However, day-long glucose and insulin responses and lipid profile were significantly reduced on the low GI diet compared with the high GI diet. Another study, which altered the GI and dietary fibre content of bread in premenopausal women with IGT and a history of gestational diabetes, showed no effect of GI on insulin sensitivity, although there appeared to be an improvement in glucose metabolism with the low GI diet [43]. In contrast, Kiens and Richter reported reduced insulin sensitivity (measured with the clamp) in healthy, lean men following a low GI, higher fibre diet compared with an isoenergetic high GI diet [44]. Though limited and based on heterogeneous groups of subjects, this evidence is contrary to the mechanistic evidence which has suggested that low GI diets might improve insulin sensitivity. In fact, low GI diets appear to reduce insulin sensitivity when measured with direct methods, such as the clamp, even though other aspects of glucose metabolism appear to be enhanced. This evidence is compatible with the findings of our study.

Few appropriately designed studies have investigated the effect of high protein (HP) diets on insulin sensitivity using direct methods of assessment. Dietary interventions comparing HP and high carbohydrate (HC) diets, designed to achieve weight loss of approximately 5% or more of total body weight, suggest that HP diets may improve glucose metabolism and insulin sensitivity in obese subjects [45,46] and those with T2DM [47,48]. However, in studies not involving weight loss, increased dietary protein intake has been associated with a reduction in insulin sensitivity and alterations in glucose metabolism that appear to be unfavourable [49,50]. An observational study showed that adults who habitually consume high protein diets (>0.8g protein/kg/day) have reduced insulin sensitivity, lower rates of glucose oxidation, greater endogenous glucose production and greater net gluconeogenesis than those consuming low protein diets (<0.8 g/kg/day) [51]. A follow-up randomised trial, comparing weight maintaining diets in overweight adults, found reduced insulin sensitivity (measured by the clamp) in those following a HP diet compared with those on a HC diet that was high in cereal fibre, after 6 weeks. However these differences were attenuated after 18 weeks, possibly as a result of decreased adherence to the HP diet [52] or effects of changes in amino acid metabolism [50]. In contrast, two recent studies comparing HP diets with either a standard protein diet matched for carbohydrate intake [53] or a HC diet [54] found no differences in insulin sensitivity after four to six weeks under weight maintenance conditions. This suggests that carbohydrate quality may be more influential with regard to insulin sensitivity than the quantity of dietary protein or carbohydrate. Nevertheless, there is mechanistic evidence which gives weight to the suggestion that excessive protein consumption could lead to impaired insulin sensitivity and glucose metabolism [49,55]. In humans an increase in plasma amino acid concentrations (by intravenous infusion) has been shown to cause a reduction in insulin-stimulated peripheral glucose uptake by inhibiting glucose transport [55,56], while cell-culture studies have also demonstrated that excess amino acids inhibit glucose transport and suppress insulin-mediated inhibition of hepatic gluconeogenesis [57]. These effects appear to be related to activation of the Mammalian Target of Rapamycin (mTOR) nutrient sensing pathway which mediates protein synthesis [58]. mTOR activates the p70 S6 kinase 1 (S6K1) pathway, which phosphorylates and inhibits the insulin receptor substrate (IRS1), resulting in an apparent reduction in insulin sensitivity. Weickert and colleagues showed that S6KI expression was increased in subjects consuming a high protein diet over 6-weeks in association with a reduction in insulin sensitivity [52].

Layman and Baum [59] propose an alternative theory: dietary protein stabilizes blood glucose concentrations when protein intakes are high. Dietary protein is more slowly metabolized than dietary carbohydrate, resulting in lower postprandial glucose and insulin responses after a high protein meal compared with a high carbohydrate meal [60,61]. Therefore while a HC diet necessitates rapid insulin responses and corresponding rapid peripheral uptake of glucose to maintain acceptable blood glucose concentrations, it follows that a HP, reduced carbohydrate diet may require a modulation of peripheral glucose uptake to maintain glucose homeostasis and prevent hypoglycaemia [59]. The brain uses glucose almost exclusively as a fuel source and a constant supply of blood glucose is critical to sustain brain activity [62]. However high concentrations of glucose are toxic and thus glucose must be tightly regulated [62]. Muscle is the principal site of insulin-stimulated glucose disposal in the body and glucose transport into skeletal muscle is the rate-controlling step in glucose metabolism [32]. The rate of glucose transport is likely to be determined by the expression and activity of proteins involved in the signalling pathways regulating the translocation of GLUT-4 from intracellular vesicles to the plasma membrane [32]. In individuals with insulin resistance, the regulation of GLUT-4 may be inappropriate in response to abnormal circulating levels of a wide range of factors, including excess free fatty acids, glucose and cytokines [32]. Glucose transport may also be regulated by metabolites that act as cellular fuel sensors such as AMPK—a protein kinase, which is regulated by changes in the cellular ratio of AMP to ATP [63]. An observational study by Harber and colleagues provided interesting evidence of the capacity of the metabolic system to adapt to changes in the nature of the fuel supply [64]. In this study, subjects were fed a very low carbohydrate diet (5% carbohydrate, 65% fat, 30% protein) for seven days. Such severe dietary carbohydrate restriction required marked metabolic adaptions to prevent hypoglycaemia. After 2 days, post-absorptive glucose concentrations were reduced from baseline. However, from day 3 until the end of the study glucose returned to normal levels, suggesting an adaptation to the to the diet had occurred in order to maintain glucose homeostasis. Using isotope dilution methods, the researchers determined that peripheral glucose uptake was decreased for the duration of the study, while glucose oxidation was reduced by 43% and the rate of non-oxidative glucose uptake (i.e., for storage as glycogen) was increased. A decrease in 24-h insulin concentrations was observed, which may have contributed to increased hepatic glucose production, by stimulating gluconeogenesis and lipolysis. 

Participants on the HPHFib diet in our study increased their absolute protein intakes as well as intakes of minimally processed, high fibre carbohydrates. Although we did not measure postprandial glucose responses, the meals consumed by those on the HPHFib would have been more slowly absorbed than StdD meals thus leading to a more stable glycaemic environment and less reliance on rapid postprandial insulin-stimulated, peripheral glucose disposal during the 10-week intervention. Therefore, on the HPHFib diet, it is conceivable that glucose transport was down-regulated to prevent hypoglycaemia [65]. Based on our observations we propose that after the intravenous glucose administration during the DISST, glucose uptake in peripheral tissues in the HPHFib group could not occur as rapidly as in the StdD group, due to a diet-induced adaptation to a slower rate of glucose arrival in the blood. In conjunction with a decrease in the rate of glucose uptake, there would be a temporary rise in plasma glucose concentrations, resulting in an increase in first phase insulin secretion (AUC_5–15_).

The fact that the HPHFib and StdD groups did not receive similar attention might be perceived to be a weakness of the main study [12]. However, the purpose of this analysis was to demonstrate how the achieved dietary changes influenced insulin sensitivity, as assessed by the DISST approach [11], rather than to argue for the benefits of a HPHFib diet.

The use of the novel DISST method to directly assess insulin sensitivity and secretion is a strength of this study. The DISST method correlates extremely well with the gold standard euglycaemic clamp and will generate diagnostic insulin sensitivity values with much greater sensitivity and specificity than values generated by crude index indices based on fasting blood parameters, such as HOMA-IR [9] and the McAuley method [10]. Although DISST has not previously been used to assess the effects of dietary change, we believe it may be a superior test to the clamp, measuring insulin sensitivity in a more physiologically representative state and providing information on insulin secretion dynamics.

## 5. Conclusions

Insulin sensitivity, measured by the novel DISST method, was reduced in overweight women at risk of diabetes following an *ad libitum* high protein, high fibre diet, compared with those following a standard *ad libitum* high carbohydrate, low fat diet, after accounting for weight loss differences. However, there was a corresponding improvement in body composition and conventional cardiometabolic risk factors, including static indices of insulin sensitivity. Therefore, we propose that dynamic insulin sensitivity indicators may reflect metabolic adaptations to usual dietary intakes for maintenance of glucose homeostasis rather than an increase in risk of diabetes, and question their validity in dietary intervention studies. Further studies are required to explain and verify these effects. The cardiometabolic benefits achieved with moderate increases to fibre and protein, without emphasis on energy reduction support the use of this approach for overweight individuals at risk of diabetes.

## Figures and Tables

**Table 1:** Baseline demographic and clinical details for all participants randomized to intervention.

	Standard Diet Group	HPHFib Group
*n*	39	44
Age (years) ^1^	39 (18–65)	44 (21–61)
Body Mass Index (BMI) (kg/m^2^) ^2^	32.3 (5.1)	32.9 (5.5)
Weight (kg) ^2^	89.1 (14.3)	87.8 (16.0)
Systolic blood pressure (mm Hg) ^2^	120 (14)	119 (14)
Diastolic blood pressure (mm Hg) ^2^	78 (8)	78 (8)
Glucose status		
Normal	36 (92.3)	38 (86.4)
Impaired glucose tolerance	2 (5.1)	6 (13.6)
Diabetes	1 (2.6)	
Menstrual Status		
Premenopausal	22 (56.4)	26 (59.1)
Post-menopausal	16 (41.0)	12 (27.3)
Hysterectomy	1 (2.6)	6 (13.6)
Smoking history		
Never smoked	24 (61.5)	31 (70.5)
Former smoker	15 (38.5)	11 (25.0)
Current smoker	0	2 (4.5)
On metformin	2 (5.1)	2 (4.5)
On lipid lowering medications	3 (7.7)	3 (6.8)
On blood pressure medications	4 (10.3	4 (9.1)
Insulin resistance ^3^	13 (33.3)	23 (52.3)
DISST Insulin resistance ^4^	31%	28%

^1^ mean (range); ^2^ mean (SD); all other values are *n* (%); ^3^ defined by the McAuley method where Gffm/I ≤ 6.3 G/mU/l; ^4^ ISI_clamp equivalent_ < 1.0 e^−2^mg/kg/(pmol/L)/min—Only calculated for participants for whom Dynamic Insulin Sensitivity and Secretion Test (DISST) data was also available at week 4 or week 10 (*n* = 35 for StdD and *n* = 39 for the relatively high protein, high fibre diet (HPHFib)).

**Table 2:** Baseline, week 4 and week 10 measures for dietary variables for the standard diet (StdD) and HPHFib groups.

	Baseline Mean (SD)	Week 4 Mean (SD)	Week 10 Mean (SD)	Within Treatment *p*-Value ^1^	Overall Effect (95% CI) ^2^	*p*-Value for Overall Effect
Energy (kJ)						
StdD	8660 (2447)	7252 (1723)	7418 (1845)	0.0081		
HPHFib	8332 (2414)	7549 (1473)	7155 (1218)	0.0064	193 (−408, 794)	0.386
Protein (% TE)						
StdD	18 (3)	21 (6)	19 (4)	0.6986		
HPHFib	18 (4)	25 (4)	24 (5)	<0.0001	5.0 (3.2, 6.8)	< 0.0001
Fat (% TE)						
StdD	32 (7)	28 (5)	30 (6)	0.4272		
HPHFib	31 (6)	25 (6)	25 (5)	0.0002	−4.5 (−6.8, −2.3)	<0.0001
Saturated fat (% TE)						
StdD	13 (4)	11 (3)	11 (3)	0.033		
HPHFib	12 (3)	8 (3)	8 (3)	<0.0001	−3.1 (−4.2, −2.0)	<0.0001
Available carbohydrate (% TE)						
StdD	45 (8)	47 (8)	46 (6)	0.8512		
HPHFib	46 (6)	45 (5)	45 (5)	0.8963	−1.7 (−4.3, 1.0)	0.211
Dietary fibre (g/day)						
StdD	24 (7)	24 (8)	22 (7)	0.2023		
HPHFib	24 (6)	33 (9)	30 (7)	0.0004	9.6 (6.0, 13.1)	<0.0001
Soluble fibre (g/day)						
StdD	11 (3)	10 (3)	10 (4)	0.2454		
HPHFib	11 (4)	14 (4)	13 (3)	0.0874	3.6 (2.0, 5.1)	<0.0001
Insoluble fibre (g/day)						
StdD	13 (4)	13 (5)	12 (5)	0.2838		
HPHFib	12 (3)	18 (5)	16 (4)	0.0006	5.0 (2.9, 7.0)	0.0001

^1^ *p*-value for difference between week 0 and week 10. ^2^ As the time x group interaction effect was not significant, a cross-sectional time-series regression model (xtreg) was used to obtain the estimate from week 4 and week 10 measures. % TE = percentage of total daily energy intake.

**Table 3:** Mean (SD) measures of body composition at baseline, week 4 ^1^ and week 10 and adjusted differences between dietary groups.

	Standard Diet	HPHFib	Difference between Groups Adjusted for Baseline Value ^2^	*p*-Value for Overall Effect
Weight (kg)				
Baseline	89.2 (14.7)	85.4 (14.8)		
Week 4	89.0 (15.2)	84.2 (14.5)	−1.3 (−1.8, −0.7)	<0.0001
Week 10	89.0 (15.1)	83.9 (14.5)	−1.1 (−1.9, −0.3)	0.006
Fat mass (kg)				
Baseline	40.9 (11.2)	39.1 (11.2)		
Week 10	41.2 (11.5)	38.1 (10.8)	−1.0 (−1.8, −0.2)	0.014
Fat mass (%)				
Baseline	45.8 (6.3)	45.6 (6.0)		
Week 10	46.2 (6.3)	45.0 (6.1)	−0.6 (−1.30, 0.02)	0.059
Truncal fat mass (kg)				
Baseline	21 (6.7)	19.9 (6.0)		
Week 10	21.3 (6.9)	19.3 (5.8)	−0.7 (−1.3, −0.1)	0.034
Lean mass (kg)				
Baseline	44.3 (5.2)	42.5 (4.8)		
Week 10	43.9 (5.1)	42.4 (4.7)	0.1 (−0.5, 0.6)	0.843
Waist circumference (cm)				
Baseline	96.6 (11.5)	94.5 (13.3)		
Week 4	95.6 (11.3)	93.2 (13.1)	−0.8 (−2.3, 0.6)	0.266
Week 10	95.8 (12.0)	92.3 (12.6)	−1.3 (−2.8, 0.2)	0.084

^1^ Dual x-ray absorptiometry (DXA) measures (fat and lean mass) were assessed at baseline and week 10 only; ^2^ A mixed model using “participant” as a random effect was used to estimate the effect of the treatment over the two intervention phases (weeks 0–4 and weeks 5–10), using baseline values as a covariate. As the time x group interaction effect was not significant, a cross-sectional time-series regression model (xtreg) was used to obtain the overall estimate from week 4 and week 10 measures.

**Table 4:** Geometric mean (min, max) DISST measures of insulin sensitivity and secretion at baseline, week 4 and week 10 and percentage differences between dietary groups adjusted for baseline values and weight change.

	Std Diet	HPHFib	Difference between Groups Adjusted for Baseline Value ^1^	*p*-Value	Difference between Groups Adjusted for Baseline Value and Weight Change ^1^	*p*-Value for Overall Effect
DISST IS (e^−4^L/pmol/min)						
Baseline	0.95 (0.33, 2.63)	0.97 (0.39, 2.61)				
Week 4	1.02 (0.38, 2.43)	0.91 (0.19, 2.47)	−9.7% (−24.2%, 7.4%)	0.245	−13.6% (−29.5%, 6.1%)	0.16
Week 10	0.98 (0.35, 3.14)	0.86 (0.25, 1.8)	−13.4% (−26.4%, 2%)	0.084	−19.3% (−31.8%, −4.5%)	0.013
Overall^1^			−12.1% (−23.5%, 1%)	0.069	−17.8% (−28.6%, −5.3%)	0.007
Basal insulin secretion, U_b_, (pmol/min)						
Baseline	215 (95, 380)	229 (94, 512)				
Week 4	205 (99, 384)	229 (109, 429)	1.3% (−6%, 9.3%)	0.727	6.4% (−2.4%, 16%)	0.154
Week 10	218 (91, 444)	206 (88, 462)	−12.1% (−20.4%, −2.9%)	0.012	−9.2% (−18.3%, 1%)	0.074
Overall					Significant time × diet effect	
1st phase AUC insulin secretion, AUC_5−15_, (pmol)						
Baseline	4913 (1164, 16037)	5788 (2193, 13638)				
Week 4	4619 (1195, 14788)	6028 (1686, 15186)	11% (−2.1%, 25.8%)	0.101	9.4% (−5.5%, 26.7%)	0.223
Week 10	4793 (1503, 13921)	5899 (2485, 16160)	6.9% (−3.8%, 18.8%)	0.209	7.2% (−4.1%, 19.9%)	0.216
Overall			9.2% (−0.5%, 19.9%)	0.064	9.2% (−1.2%, 20.7%)	0.084
2nd phase AUC insulin secretion, U_2nd_, (pmol)						
Baseline	6143 (1588, 12350)	6388 (2750, 15932)				
Week 4	5919 (1850, 12631)	6470 (2269, 17434)	1.9% (−8.3%, 13.3%)	0.719	5% (−7.2%, 18.9%)	0.433
Week 10	6088 (1933, 13892)	5969 (2238, 19210)	−6% (−16.5%, 5.7%)	0.297	−6.6% (−17.9%, 6.2%)	0.29
Overall			−2% (−11.2%, 8.1%)	0.688	−1.8% (−11.9%, 9.3%)	0.734
Total insulin secretion, U_total_, (pmol/L)						
Baseline	15266 (7175, 27275)	16456 (6793, 37618)				
Week 4	14538 (7156, 25774)	16927 (8061, 39962)	5.4% (−1.6%, 12.9%)	0.133	7.1% (−1.2%, 16.2%)	0.096
Week 10	15102 (7234, 27159)	15835 (6231, 43324)	−3% (−10%, 4.4%)	0.411	−2.9% (−10.4%, 5.3%)	0.473
Overall					Significant time*diet effect	

^1^ A mixed model using “participant” as a random effect was used to estimate the effect of the treatment over the two intervention phases (weeks 0–4 and weeks 5–10), using baseline values as a covariate. If the time x group interaction effect was not significant, a cross-sectional time-series regression model (xtreg) was used to obtain the overall estimate from week 4 and week 10 measures. AUC: area under the curve.

**Table 5:** Geometric mean (min, max) measures of insulin sensitivity, based on fasting blood samples at baseline, week 4 and week 10 and percentage differences between dietary groups, adjusted for baseline values and weight change.

	Standard Diet	HPHFib	Difference Adjusted for Baseline Value ^1^	*p*-Value	Difference Adjusted for Baseline Value and Weight Change ^1^	*p*-Value for Overall Effect
Fasting plasma glucose (mmol/L)						
Baseline	4.7 (3.8, 8.1)	4.7 (4.1, 5.7)				
Week 4	4.7 (3.6, 6.4)	4.6 (3.8, 5.9)	−0.5% (−3.3%, 2.4%)	0.74	1.2% (−2%, 4.5%)	0.449
Week 10	4.8 (3.9, 6.1)	4.6 (3.8, 5.8)	−3.8% (−6.7%, −0.8%)	0.014	−3.1% (−6.3%, 0.1%)	0.057
Fasting plasma insulin (pmol/L)						
Baseline	61.2 (18.8, 174.3)	77.5 (11.8, 729.9)				
Week 4	57.0 (13.2, 206.3)	66.1 (13.9, 190.3)	−4.1% (−20.2%, 15.2%)	0.649	4.7% (−15.1%, 29.1%)	0.664
Week 10	59.8 (20.8, 197.9)	63.4 (9, 206.3)	−10% (−26.6%, 10.2%)	0.302	−2.1% (-21%, 21.3%)	0.844
McAuley IS index						
Baseline	7.26 (3.3, 10.26)	6.69 (3.38, 14.23)				
Week 4	7.45 (3.6, 13.95)	7.15 (4.61, 11.89)	4.2% (−3.6%, 12.6%)	0.295	0.4% (−8.1%, 9.6%)	0.932
Week 10	7.20 (3.55, 12.81)	7.30 (3.85, 15.26)	8.5% (−0.1%, 17.9%)	0.053	5.3% (−3.6%, 15%)	0.245
HOMA−IR Index						
Baseline	1.28 (0.39, 3.69)	1.60 (0.24, 12.35)				
Week 4	1.19 (0.28, 4.18)	1.37 (0.29, 3.79)	−4.4% (−20.2%, 14.5%)	0.621	4.4% (−15.1%, 28.3%)	0.68
Week 10	1.26 (0.44, 4.08)	1.31 (0.18, 3.98)	−11.2% (−27.3%, 8.4%)	0.24	−3.8% (−22.1%, 18.8%)	0.714

^1^ A mixed model using “participant” as a random effect was used to estimate the effect of the treatment over the two intervention phases (weeks 0–4 and weeks 5–10) using baseline values as a covariate. As the time × group interaction effect was not significant, a cross-sectional time-series regression model (xtreg) was used to obtain the overall estimates from week 4 and week 10 measures; IS: Insulin sensitivity; IR: Insulin resistance.

**Table 6:** Mean (SD) measures for other metabolic variables based on fasting blood samples and clinical measures at baseline, week 4 and week 10 and percentage differences between dietary groups, adjusted for baseline values and weight change.

	Standard Diet	HPHFib	Difference Adjusted for Baseline Value ^1^	*p*-Value	Difference Adjusted for Baseline Value and Weight Change ^1^	*p*-Value
Total cholesterol (mmol/L)						
Baseline	4.87 (1.45)	4.57 (0.82)				
Week 4	4.92 (1.58)	4.32 (0.82)	−0.28 (−0.51, −0.04)	0.021	−0.25 (−0.48, −0.02)	0.031
Week 10	5.03 (1.62)	4.36 (0.79)	−0.40 (−0.66, −0.15)	0.002	−0.36 (−0.63, −0.09)	0.01
LDL cholesterol (mmol/L)						
Baseline	2.94 (1.00)	2.8 (0.74)				
Week 4	2.92 (0.89)	2.69 (0.77)	−0.11 (−0.3, 0.09)	0.274	−0.15 (−0.37, 0.07)	0.173
Week 10	3.00 (0.98)	2.66 (0.71)	−0.25 (−0.48, −0.03)	0.029	−0.23 (−0.47, 0.01)	0.065
HDL cholesterol (mmol/L)						
Baseline	1.13 (0.29)	1.19 (0.34)				
Week 4	1.10 (0.28)	1.09 (0.28)	−0.07 (−0.13, −0.02)	0.008	−0.07 (−0.13, −0.01)	0.032
Week 10	1.12 (0.29)	1.17 (0.34)	−0.02 (−0.09, 0.05)	0.534	−0.02 (−0.1, 0.05)	0.486
Triglycerides (mmol/L)						
Baseline	1.42 (1.45)	1.32 (0.60)				
Week 4	1.37 (0.95)	1.21 (0.51)	−0.09 (−0.28, 0.1)	0.351	−0.1 (−0.3, 0.09)	0.295
Week 10	1.42 (1.03)	1.21 (0.68)	−0.14 (−0.38, 0.11)	0.272	−0.09 (−0.35, 0.17)	0.506
Systolic BP (mmHg)						
Baseline	120.7 (14.2)	118.7 (15.0)				
Week 4	124.1 (13.9)	117.0 (15.9)	−3.8 (−11.0, 3.5)	0.299	−5.1 (−13.4, 3.1)	0.217
Week 10	121.9 (15.0)	117.1 (12.8)	−2.0 (−7.4, 3.4)	0.469	−1.5 (−7.2, 4.3)	0.607
Diastolic BP (mmHg)						
Baseline	77.8 (8.3)	78.2 (8.1)				
Week 4	76.1 (8.1)	75.2 (10.2)	−0.78 (−5.3, 3.7)	0.972	−1.1 (−6.0, 3.8)	0.641
Week 10	75.7 (8.1)	74.8 (8.0)	−0.27 (−3.6, 3.1)	0.875	−0.1 (−3.6, 3.5)	0.972

^1^ A mixed model using “participant” as a random effect was used to estimate the effect of the treatment over the two intervention phases (weeks 0–4 and weeks 5–10), using baseline values as a covariate. As the time × group interaction effect was not significant, a cross-sectional time-series regression model (xtreg) was used to obtain the overall estimates from week 4 and week 10 measures; BP: Blood pressure.
